# Correlates of household waste management in Ghana: implications for public health

**DOI:** 10.1016/j.heliyon.2021.e08227

**Published:** 2021-10-21

**Authors:** Stephen T. Odonkor, Anthony M. Sallar

**Affiliations:** a School of Public Services and Governance, Ghana Institute of Management and Public Administration Accra, Ghana; b School of Liberal Arts and Social Sciences, Ghana Institute of Management and Public Administration Accra, Ghana

**Keywords:** Floods, Waste management, Public health, Pollution, Disaster

## Abstract

Household waste management is a challenge in Accra, Ghana, due to increasing urbanization and unscrupulous dumping of garbage. The aim of this study was to determine the correlates of household waste management as well as their implications for public health. The study employed a descriptive, cross-sectional design with self-administered questionnaires to assess household waste management in Accra, Ghana. The study was conducted from September 1, 2019 to February 28, 2020. Our findings showed that rubber waste types were the most generated (26%) among respondents in their various households, followed by tin (19%) and plastic (16%). Majority (50.5%) of the respondents disposed of waste collected in public bins. However, about half of the respondents dumped waste through illegal or unauthorized means. About three out of four respondents (72.9%) indicated that improper management of garbage could affect the health of household members while 81.1% reported willing to participate in waste management in their neighborhoods. Females were more likely to be willing to participate in waste management compared to their male counterparts (p = 0.001). It is recommended that stakeholders and policy makers should focus on education for the citizenry on waste management behaviors. In addition, they should have increased access to waste collection services; since the public health implication of dumping anywhere could cause flooding, choke gutters, and lead to epidemic of cholera and vector borne diseases like malaria and dysentery.

## 1 Introduction

Wastes in the form of solids liquids or gases, can be generated in nearly all working environments. Based on their sources, wastes can be classified as household/domestic, industrial, agricultural, commercial, mining and construction waste (Amasuomo and Baird, 2016). Amounts of waste produced from these sources vary due to differences in the nature of operations or activities. For example, waste generation from the construction industry are fast rising and according to Solís-Guzmán et al. (2009), it accounts for about 35% of the total waste generated worldwide. Construction wastes usually cause physical damage to the environment and as a result, significant financial losses can be incurred in fixing it (Mah et al., 2018). Industrial waste mainly consists of toxic and hazardous substances that can cause both injuries and health complications when they come into contact with humans. Additionally, hazardous and toxic wastes are also vital facilitators of climate change as they emit considerable amounts of greenhouse gases into the atmosphere (Nnaji and Utsev, 2011). The extent to which the health effects and dangers associated with waste would be alleviated depend on the effectiveness of waste management protocols available.

According to Boadi et al. (2005) many unforeseen problems have come about in many African cities because of increasing urbanisation in the cities. These cities have been confronted with socio economic problems such as poverty, unemployment, and related social vices like commercial sex and crime. Furthermore, causes of death in major urban centres could be attributed to prevalence of infectious diseases such as poor sanitation, inadequate portable water and air pollution.

It is almost inevitable to completely eradicate waste, as the actions or undertakings of humans are constantly leading to waste production (Brunner and Rechberger, 2015). There has been a considerable increase in waste generated by humans since the industrial revolution began around the 16^th^ century as compared to the periods before that (Wilson, 2007). The World Bank reports that annually the municipal solid waste generated worldwide could be around 2.01 billion tons. It was further indicated that developed nations like USA, Canada and other countries of the European Union are responsible for 34% of the total worldwide waste generation (Sáez and Osmani, 2019). In the developing world which includes Sub Saharan Africa, waste generation is comparatively low to that of the developed world. However, solid liquid and gaseous waste are increasingly becoming a problem in Africa due to urbanization (Yoada et al., 2014). According to World Bank, current magnitude of waste generation in Sub Saharan Africa is projected to triple by 2050 (Kaza and Yao, 2018).

Even though developed nations generate 34% worldwide waste, they are better managed than in developing or least developed countries (Bundhoo, 2018). In a developed nation like Israel, there are over 15 modernized and advanced landfill sites in addition to recycling facilities which are capable of recycling an estimated 23% of the country's total waste generation (IMER, 2010).

Comparative analysis shows that waste management differences in developed and developing countries can partly be attributed to the composition of their respective municipal solid waste. For instance, household waste in middle to low-income countries usually contains over 50% organic substances as opposed to that of high-income countries which contain less than 30% organics (Khatib, 2011). Organic waste is difficult to manage due to the fact that, they easily decay, moisturize and give off bad odour. In this regard, household waste management tends to be less challenging in developed nations as compared to third world countries. Otieno (2010) reported that 30%–40% of solid waste generated in Kenya's urban settings were unattended to and less than half of the country's urban population received the services of waste management agencies. In Ghana, Gbeddy (2018) investigated municipal waste collection system in the Municipality of Accra through an online survey. His findings revealed that the quality of waste management in the municipality was of utmost concern to 83% of participants. They reported that waste collectors were inconsistent, unprofessional and delayed in arrival among several others.

Lack of proper dumping sites in developing and least developed nations also play a significant role in the uncontrolled disposal of waste, consequently posing high risks to the health of urban dwellers (Boadi and Kuitunen, 2005). In Africa, municipal solid waste in most urban dwellings are collected and disposed off at unregulated erratic dump sites, burnt or dumped in water bodies (Aryampa et al., 2019; Khatib, 2011) all of which have implications for public health. This indiscriminate dumping of waste and consequent clogging of gutters has become a perennial flooding problem in the big cities especially Accra. The result was a tragic accident on June 4^th^ 2015 when an explosion at a GOIL fuel filling station resulting into the deaths of about 150 people (BBC, 2015). The victims were commuters who were trying to escape the massive downpour and took shelter at the station when flood water swept stored oil towards a fire and resulted into an inferno.

Solid waste from households provides breeding grounds for mosquitoes and flies which are vectors of malaria, dengue fever and cholera and rats which are vectors of rodent borne diseases (Nor Faiza et al., 2019). In addition, Boadi et al. (2005) found an association between storing waste at home and the presence of houseflies in the kitchen in their study on household-level waste management in Accra. The presence of houseflies was also correlated with the outbreak of child-related diarrhea. Though waste -related diseases and dangers are globally endemic, its incidence in middle-low-income countries, including Africa have not been recognized (Landrigan et al., 2015).

Generally, waste management in Africa is receiving widespread recognition in recent times as systems of waste disposal in several countries have emerged but information on the correlates of such procedures is seriously lacking. According to Aryampa et al. (2019), drivers of the waste management process are as important as the process itself in the attainment of sustainability. They postulated that increasing research on the drivers of household waste management was relevant in coming up with strategic interventions on how to successfully integrate waste management into socioeconomic development. Aryampa et al. (2019) contended that would be beneficial in several aspects. Firstly, financial resources directed towards waste management would be managed properly. Secondly, it will build a solid foundation for exploiting employment opportunities and income generation at both individual and national levels. Finally, household or municipal waste management will improve and human health would be less threatened. In Accra, Ghana, which is the current study's location, household waste generation is on the rise due to increasing urbanization, rapid population growth, and inability of the government to constantly provide the required financial resources to companies involved in waste disposal.

It is worth noting that like in most developing countries, municipal solid waste management in Ghana's capital is poor. In Accra, the private sector has been responsible for municipal solid waste management for over 20 years. However, waste collection coverage stands at 75% while 62% of the total waste generated are deposited at regulated landfills (Oduro-Appiah et al., 2017). Thus, a significant amount of waste produced in the city is deposited erratically either by the general public or unauthorized waste dealers in the environment which further aggravates human health risks. The participation of household members in municipal waste management in Accra and Ghana as a whole has not been extensively studied. These include categories of waste generated, methods of disposal, waste management problems in the neighborhood, and willing to participate in waste management. The aim of this study was to examine the correlates of household waste management in Accra as well as their implications for public health. Having substantial data on past and present waste generation levels would be a significant step in attaining sustainable development because associated problems and opportunities could be exploited for future improvements (Mohee and Simelane, 2015).

## 2 Materials and methods

### 2.1 Study site description

**Figure 1:**
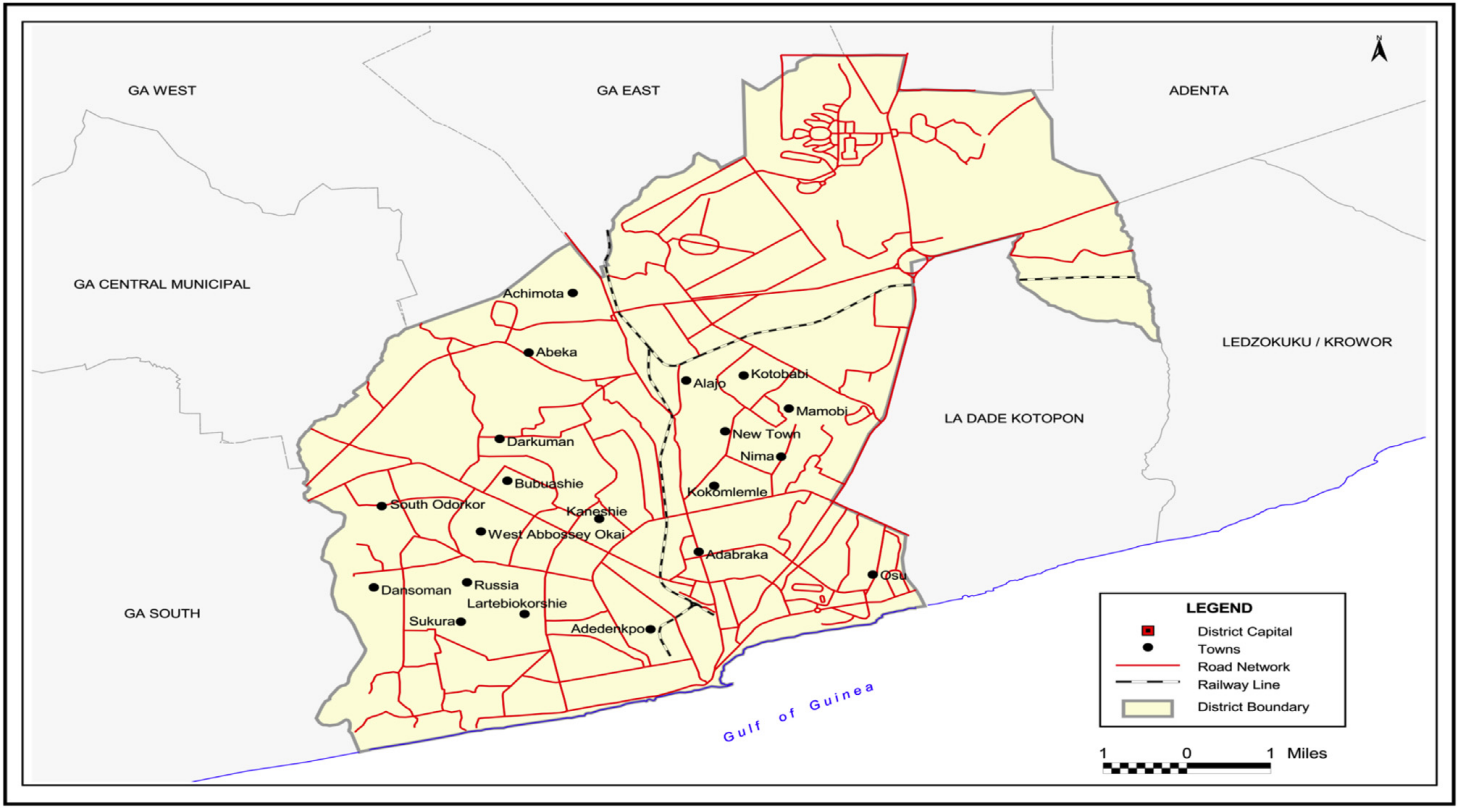
Map of Accra.

The study was done in Accra (Figure 1), the national capital of Ghana. Accra has a population growth rate of 3.1 % per annum. It is a very active and vibrant city, which experiences huge immigration from other regions within the country and other countries in the West African sub region.

### 2.2 Research design and sample size

In this study, we unitized a descriptive, cross-sectional design. Questionnaires were self- and in-person administered. The study was carried out from the 1^st^ of September 2019 to 28^th^ February 2020. We utilized Miller and Brewer's (2003) mathematical formula to determine the sample size It took about 28 min on average to complete a questionnaire.

### 2.3 Sampling technique

Data were obtained from a representative survey of households (N = 200). A stratified sampling technique was used to obtain the required number of respondents from households within the three (3) demarcated zones of the city: southern; middle; and northern belts.

### 2.4 Respondents consent and data collection

Before respondents were interviewed, we collected verbal as well as written informed consents from them. We informed them about the objectives of the study as well letting them know that taking part was voluntary. They were further assured that they could opt out of the study and nothing would be done to them and the social status or respect they had in the community would not be affected. They were assured of confidentiality that no one would know of their identity as well as their responses. To ensure confidentiality promised to the respondents, we removed any identifier that could be traced to any respondent. We sought and obtained ethical clearance for this study from Ethics Review Committee of the Ghana Institute of Management and Public Administration.

Data was collected from a representative household survey of those resident in Accra. Before embarking on pretesting, we submitted our questionnaire initially to a group of individuals who were experts in the discipline for review as pertains to content and face validity. Recommendations were made and were incorporated into the questionnaire before pilot testing. We trained five research assistants to assist in the questionnaire administration. This was because some of the respondents were not be able to read in the English language and if the research assistants only translated at the time of the interview, they might have introduced bias by each individual's translation. This would require the research assistants translate into the 2 local languages (Ga and Twi). To minimize or eliminate bias due to individual translation and to achieve consistency in administration, the questionnaires were translated in the local dialects and back translated into English to determine whether the understandings were consistent. This was done to ensure that the differences in responses were due to the respondents and not because of the way the questions were asked or different meanings were attached to them. A further test was done on key variables on a population that was not in the study. This enabled us to compute a reliability test from English administration of the questionnaire and local dialect of the questionnaire on the same population few days after the first administration (similar to test-retest reliability). The final instrument was pilot tested on 50 citizens in one of the regional capitals in Ghana who were not involved in the study for reliability of the instrument. The internal consistency for reliability coefficients for category of waste generated, disposal site of waste collected, and waste management problems of the instrument respectively yielded Cronbach alpha of 0.78, 0.83, and 0.85 which we considered high enough and met our criteria.

### 2.5 Data analysis

Descriptive statistics (frequencies, means and standard deviations whenever appropriate) were used to describe some demographic characteristics and responses to the questions. The chi-square test was used to test the differences between males and females with regard to the outcome variable of willingness to participate in waste management and statistical test of alpha = 0.05 or less was considered statistically significant**.**

## 3 Results

### 3.1 Demographic characteristics of respondents

A total of 220 questionnaires were distributed for the study. However, 200 were completely filed and returned, which represents a 90.9% response rate.

Table 1 presents the sociodemographic characteristics of the respondents. Female respondents constituted 76%. About 2 out of 5 respondents (41%) were between the ages of 31–40 (41%) while the 12.5% were those aged 51 years and over**.** Almost two out of three respondents (56.5%) were married and 13% of the respondents had no formal education.

**Table 1:** Socio-demographic data of respondent.

Variable (N = 200)	Number(N)	Percentage (%)
**Gender**		
Female	152	76.0
Male	48	24.0
**Age**		
20–30	44	22.0
31–40	82	41.0
41–50	49	24.5
≥51	25	12.5
**Religion**		
Christianity	153	76.5
Islamic	47	23.5
**Residence**		
Urban	179	89.5
Rural	21	10.5
**Marital Status**		
Single	73	36.5
Married	113	56.5
Divorced/separated	14	7.0
**Educational level**		
No-formal	26	13.0
JHS/SHS	67	33.5
Tertiary	107	53.5
**Employment status**		
Employed	130	65.0
Unemployed	70	35.0
**Family size**		
1–5	124	62.0
6–10	63	31.5
≥11	13	6.5
**Income Level**		
High	72	36.0
Middle	80	40.0
Low	48	24.0

### 3.2 Category of waste generated and methods of disposal

**Figure 2:**
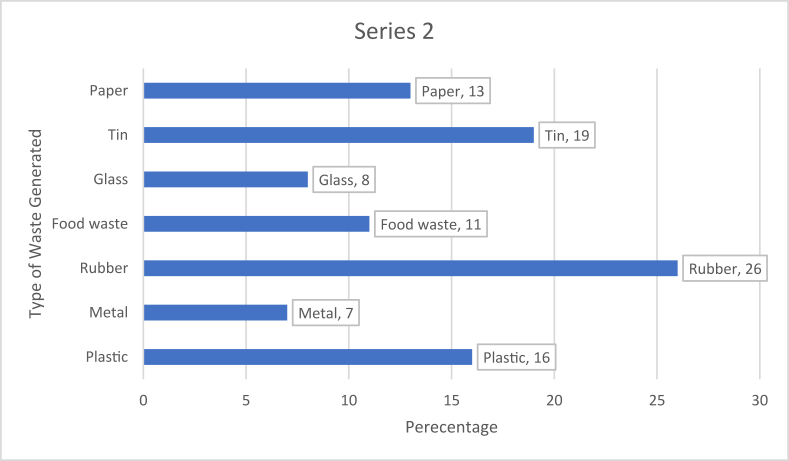
Category of waste generated by Households.

Figure 2 shows the category of waste often generated by the households of respondents. It was observed that, rubber waste types were the most generated (26%) among respondents in their various households, followed by tin wastes (19%). Metallic wastes were the least generated (6%) among the households.

Table 2 shows data on sites respondents disposed waste generated from their households. Majority (50.5%) of the respondents disposed waste collected in public bins. In addition, 16.5% and 14.5% of the respondents admitted they dumped collected waste into gutters and water bodies, respectively.

**Table 2:** Disposal site of waste collected.

Variable	Number (N)	Percentage (%)
Gutter	33	16.5
Open area	18	9.0
Water body	29	14.5
Public bin	101	50.5
Burn	19	9.5
**Total**	**200**	**100**

As can be seen in Table 2, 30% (16.5% plus 14.5%) of the garbage that is disposed in areas where it can cause flooding after the rains. As regards who was tasked with household waste disposal 55% of the respondents indicated that children dispose of the collected waste, waste disposal contractors (28%) were paid to dispose of house hold waste. Adults (17%) were last group of people who disposed of household waste.

### *3.3* Reported waste management problems

In Table 3, respondents' opinions on waste management problems in their neighborhoods are shown. Data analyzed on participants’ responses revealed each one of the enlisted variables of waste management problems was a significant challenge in the neighborhoods. However, vector borne diseases were indicated by 22% of the respondents as a problem of waste management while the least (9.5%) indicated water shortage or drought.

**Table 3:** Reported waste management problems in the neighborhood.

	Waste problems in the neighborhood	
	Yes N (%)	No N (%)
Land pollution	21 (10.5)	6 (3.0)
Respiratory problem	32 (16.0)	3 (1.5)
Vector-borne disease	44 (22.0)	4 (2.0)
Water shortage/Drought	19 (9.5)	8 (4.0)
Contaminated water	30 (15.0)	5 (2.5)
Flood	24 (12.0)	4 (2.0)
**Total**	170 (85.0)	30 (15.0)

### 3.4 Relationship between waste management problems and health of household members

Most of the respondents (61.9%) who reported the presence of waste management problems in their neighborhoods indicated that these problems affected the health of households (Table 4). Also, 11% of respondents who said there were no waste management problems in their neighborhoods linked improper waste disposal practices to the health of household members. Only 0.6% of the respondents reported no waste management problems in their neighborhoods, indicated there was no relationship between such problems and the health of household members.

**Table 4:** Waste management problems and health of household members.

Variable	Waste management problems in the community		Total N (%)	Significance
	Yes N (%)	No N (%)		
Improper waste management affects the health of household members				
Yes	119 (61.9)	2 (0.6)	121 (62.5)	X^2^ = 36.604
No	51 (26.5)	28 (11.0)	79 (37.5)	P = 0.001
Total	170 (88.4)	30 (11.6)	200 (100)	Df = 1

### 3.5 Willingness to participate in waste management

The willingness of the respondents to participate in waste management is presented in Table 5. Generally, it was observed that more than four out of five respondents (81.1%) were willing to participate in waste management. Among this the percentage of female respondents (41.8%) was higher than the male respondents (39.3%). Females were more likely to be willing to participate in waste management compared to their males’ counterparts (p = 0.001).

**Table 5:** Willingness to Participate in waste management.

Variable	Gender of respondent		Total N (%)	Significance
	Female (%)	Male (%)		
Do you want to be involved				
Yes	118 (41.8)	41 (39.3)	159 (81.1)	X^2^ = 7.36
No	34 (12.3)	7 (6.6)	41 (18.9)	P = 0.001
Total	152 (54.1)	48 (45.9)	200 (100)	

## 4 Discussion

Findings of the present study will contribute to already available literature on household waste management. Specifically, the study determined the various categories of household generated waste and methods of their disposal; reported waste management problems and their link to health of household members and finally respondents’ willingness to participate in household waste management and related drivers. Such information is vital in policy making and directions towards effective municipal waste management in the Accra municipality.

### 4.1 Demographics

About three out of four respondents (76%) were females. Even though this may likely introduce bias it is less likely since in our finding respondents reported that children were responsible for waste disposal (55%) and contractors (28%) and adults only 17%. A study by Dalen and Halvorsen (2011) involving 10,000 participants reported that the female respondents were more positive to hypothesized environment-related policy measures even though this might not translate into actual practices. Most of the respondents in our study had family size between 1 – 5 which was similarly reported by a related study by Adu-Boahen et al. (2014) in Ghana. This finding concurs with the national average household size of 4.0 revealed by the Ghana Living Standard Survey (Ghana Statistical Service, 2014). In terms of income levels, most of the respondents were middle class earners. Several studies have reported the significant influence of family size and income levels on household waste generation and management (Rawat and Daverey, 2018; Suthar and Singh, 2015; Grover and Singh, 2014). Grover and Singh (2014) reported a positive correlation between family size and residential waste generation among households in Dehradun, India. Suthar & Singh (2015) revealed higher household waste generation among high income households followed by the middle income and low-income household in their study. This is not surprising because the more economically endowed one is, the likely possibility of spending more and hence creating more waste compared to a poor person eking subsistence living. According to Khatib (2011). household waste generation in middle to low-income countries which includes Ghana is mainly composed of organic substances (over 50%). However, in our study, we observed that food waste made up only 11% while rubber waste dominated with 26% of total municipal waste generation and this has implications for public health.

### 4.2 Plastic waste generation

In our study plastic waste generation constituted 16%. However, in another study in Ghana, plastic made up 64.3% of total waste produced among household in the Madina Municipality in Accra (Yoada et al., 2014), which is one of the most densely populated area in Ghana. This calls for the need for extensive location specific research for a better overview of waste generation in developing nations and it is imperative developing countries develop workable strategies to combat it. Additionally, we identified plastics as the third highest waste product from the households which supports the reported increasing levels of plastic generation in urban dwellings of Africa (Godfrey, 2019). Having rubber and plastics as components of household trash have serious implication for waste management and potential hazards. For instance, plastic waste is mostly associated with industries and as a result it is gradually becoming a major part of household garbage. It is likely to intensify the emission of toxic substances in the atmosphere especially when they are burnt and it can also clog drainage systems hence contributing to flooding. Waste generated because of plastic bags are responsible for clogging oceans, killing marine life and considered a major global issue which situation is worsened because it can take hundreds of years to degrade and. The authorities of Rwanda said contended that bags made of plastic contribute to flooding and prevent crops from growing because rainwater can't penetrate the soil when it is littered with plastic hence consequently banned their use in 2008. An exemption to the ban involved specific industries such as hospitals and pharmaceuticals (BBC, 2019). Penalties ranged from smugglers receiving sentences maximum of six months in jail and the executives of business establishments involved in manufacturing or storing plastic bags could be imprisoned maximum of a year. As part of the enforcement measures, shop owners who were found guilty for wrapping bread in cellophane were shut down, fined, and the owners wrote and signed apology letters— all as part of the nation's environmental cleanup (BBC, 2019). Similarly, in 2017 Kenya passed a law banning the use of plastic bags, its manufacturing, sale and distribution with penalties of up to $40,000 or (£32,000) or be jailed up to four years (BBC, 2019). An earlier study, supported by its National Environmental Management Agency (NEMA), reported that one of every two (50%) cattle in its urban areas had plastic in their stomachs.

### 4.3 Illegal waste disposal

According to Ghana's National Waste Management Guidelines waste should be disposed of at land-filled sites and not deposited or scattered on the surface of open dumps. This is in consonance with the literature which indicated that household generated refuse (solid wastes) if not properly managed may create routes for transmission of diseases (Adama and Esena, 2016). In spite of the above our results indicate significant amounts of garbage is dumped in unauthorized places including the bushes, water bodies, gutters, on the streets, among several other locations in the open environment, which is consistent with observations made by Yoada et al. (2014). This behavior is likely to increase the proliferation of vector borne diseases and threaten general public health. The reality is that people who do the right thing with regard to waste management are equally at risk. In our findings, we noted that nearly half of the respondents disposed waste through illegal or unauthorized means even though the other half employed the services of public bins. Specifically, 49.5% of respondents got rid of waste generated from their households in gutters, open areas, water bodies or by burning. Raw open dumping of garbage was one of the main causes of the devastating cholera outbreak that occurred in Accra in 2014/2015 (Ohene-Adjei et al., 2017). Another wave of this outbreak could happen if steps are not taken to alleviate open dumping of waste in Accra. Furthermore, children could be at elevated risks of cholera infection and other diseases considering that, there were largely identified to be the household waste disposers, in the present study. It is important to state that waste disposal is not the responsibility of children in Ghana but that of the Ministry of Local Government and Rural Development (Norley, 2009).

Poor waste management endangers the environment and human lives (Addy, 2013). Respondents identified land pollution, respiratory problems, vector-borne diseases, water shortage, contaminated water and flood as significant waste management problems in their neighbourhoods. This finding is probably due to the revealed poor waste disposal methods among the respondents. It is often being argued that knowledge of the impacts of an environmental problem does not necessarily translate into positive attitude or good practices towards it (Kightley et al., 2013; Dalen and Halvorsen, 2011). In our study the way respondents perceived the relationship between waste management and health of household members (72.9%) indicated that they were aware improper management of garbage could affect the health of household numbers despite the fact that nearly half of them did not engage in good waste disposal practices.

In our study, 81.1% were willing to participate in waste management which is consistent with related studies in Nigeria and Uganda where 84.2% and 76,6% of household members respectively were willing to participate in waste management (Miner et al., 2020; Mukama et al., 2016). Furthermore, in these studies, the respondents reported poor waste handling and disposal practices. Based on this, educating households in the Accra Municipal on the health ramifications associated with garbage may not be highly effective in alleviating waste anti management practices in the capital. Perhaps, strategizing educational campaigns such that it brings out positive attitudes and practices in residents towards waste disposal could be a better alternative.

We believe that our findings give credence to what Boadi et al. (2005) suggested that jobs could be created using recycling to turn huge waste into useful resources. Recycling does not only create jobs but can also improve the environment. The reason is that it would reduce indiscriminate waste disposal, would decrease the quantity of waste to be disposed of in landfills, and depletion of resources. For example, aluminum could be recovered and sold to small-scale recyclers to produce valuable items such as lamps and cooking utensils to compete with imports. There is the need to reinforce attitudes which include recycling of wastes such as glass, metals and plastics, which would lead to decrease the amount of waste to be sent to landfills. It is also imperative to make people through environmental health awareness programmes see the link between poor sanitation and hygiene, and physical wellbeing (Boadi et al., 2005).

### 4.4 Study limitation

Since it is a cross-sectional study hence it analyzed information at a particular point in time and has shortcomings associated with such studies. For example, it is likely with changes to new information, the participants might possibly respond differently to issues of garbage waste and disposal.

Since the study was done in Accra the capital city of Ghana, we could possibly have introduced bias in our results because the density of the population could result in different responses if the study were conducted in the rural areas.

Since only those who are surveyed and those willing to participate is an attribute of cross-sectional studies, it can introduce volunteer bias. Furthermore, issues of temporality which is one of the main shortcomings of cross-sectional study may be present. Despite these, cross sectional studies such as this are important because it has provided relationship between factors that impact on waste disposal which is contributory factors to flooding, vector borne diseases and overall sanitation in the capital city. Furthermore, it provided us with a snapshot of the data which can be used for intervention as well as for planning of health services, future ideas for legislation relating to bans on plastic waste as has been successfully done in other countries.

## 5 Conclusion

In the present study, we noted that rubber, tins and plastics were the main constituents of waste generated by the households in Accra. Secondly, about half of the respondents engage in illegal waste disposal practices while the other half utilized public bins. Thirdly, most respondents admitted poor waste management could affect health of household members. Finally, most of the respondents indicated they were willing to participate in waste management. Based on these results, it is recommended for an effective waste management in Accra, much resources and policies should be directed at instilling positive attitudes in residents. To do this, a significant step is when policy makers and major stakeholders demonstrate the business potential of waste. Management. Support systems should be put in place that would make it possible for people, especially the unemployed, to start waste management as a source of income generation. Additionally, the city of Accra needs state-of-art recycling facilities that can manipulate all kinds of waste into useful products. When these interventions are available, waste will be regarded as a product of value by recyclers, waste collectors and even the households. This will minimize the dumping of garbage into the open environment with its potential for diseases that can results in poor sanitation.

## Declarations

### Author contribution statement

Stephen T. Odonkor: Conceived and designed the experiments; Performed the experiments; Analyzed and interpreted the data; Contributed reagents, materials, analysis tools or data; Wrote the paper.

Anthony M. Sallar: Performed the experiments; Analyzed and interpreted the data; Contributed reagents, materials, analysis tools or data; Wrote the paper.

### Funding statement

This research did not receive any specific grant from funding agencies in the public, commercial, or not-for-profit sectors.

### Data availability statement

Data included in article/supplementary material/referenced in article.

### Declaration of interests statement

The authors declare no conflict of interest.

### Additional information

No additional information is available for this paper.
